# Numerical Simulation and FRAP Experiments Show That the Plasma Membrane Binding Protein PH-EFA6 Does Not Exhibit Anomalous Subdiffusion in Cells

**DOI:** 10.3390/biom8030090

**Published:** 2018-09-05

**Authors:** Cyril Favard

**Affiliations:** Membrane Domains and Viral Assembly, IRIM, UMR 9004 CNRS—Université Montpellier, 1919, Route de Mende, 34 000 Montpellier, France; cyril.favard@irim.cnrs.fr

**Keywords:** anomalous diffusion, fluorescence recovery after photobleaching (FRAP), numerical simulations, pleckstrin homology (PH)-domain, membrane binding

## Abstract

The fluorescence recovery after photobleaching (FRAP) technique has been used for decades to measure movements of molecules in two-dimension (2D). Data obtained by FRAP experiments in cell plasma membranes are assumed to be described through a means of two parameters, a diffusion coefficient, D (as defined in a pure Brownian model) and a mobile fraction, M. Nevertheless, it has also been shown that recoveries can be nicely fit using anomalous subdiffusion. Fluorescence recovery after photobleaching (FRAP) at variable radii has been developed using the Brownian diffusion model to access geometrical characteristics of the surrounding landscape of the molecule. Here, we performed numerical simulations of continuous time random walk (CTRW) anomalous subdiffusion and interpreted them in the context of variable radii FRAP. These simulations were compared to experimental data obtained at variable radii on living cells using the pleckstrin homology (PH) domain of the membrane binding protein EFA6 (exchange factor for ARF6, a small G protein). This protein domain is an excellent candidate to explore the structure of the interface between cytosol and plasma membrane in cells. By direct comparison of our numerical simulations to the experiments, we show that this protein does not exhibit anomalous diffusion in baby hamster kidney (BHK) cells. The non Brownian PH-EFA6 dynamics observed here are more related to spatial heterogeneities such as cytoskeleton fence effects.

## 1. Introduction

Early models of the plasma membrane, notably the fluid mosaic model [1], postulated that transmembrane proteins were freely diffusing in a sea of lipids. During these two last decades, it has become apparent that cell surface membranes are far from being a homogeneous mixture of their lipid and protein components. They are compartmented into domains whose composition, physical properties and function are different. Numerous studies on transmembrane proteins and plasma membrane lipids by means of single particle tracking (SPT), fluorescence correlation spectroscopy (FCS) or fluorescence recovery after photobleaching (FRAP) have shown the existence of micro and nanometer size domains on both model membrane [2,3] and living cells [4,5,6]. In the plasma membrane of living cells, these domains can come from different origins but are generally classified into two main groups:

- “Rafts” model where lipid/lipid phase separation drives the lateral partitioning of transmembrane proteins [7].
- “Cytoskeleton fence” model in which transmembrane proteins are coralled by a fence of cytoskeleton just beneath the plasma membrane [8,9].

First, variable radii FRAP [5,6], and then spot variation FCS [10,11,12] helped in discriminating amongst these two models the nature of the deviation to pure Brownian diffusion of membrane components in living cells.

Fluorescence recovery after photobleachingexperiments have been used for determination of long-range molecular diffusion of proteins and lipids on both the model system and cells for more than 30 years [13,14]. Briefly, fluorescently labelled molecules localized within a predefined area are irreversibly photodestructed by a short and intense laser pulse. The recovery of the fluorescence in this area is then measured against time. Since no reversible photoreaction occurs, recovery of the fluorescence in the photobleached area is due to diffusion. Fluorescence Recovery After Photobleaching data are generally interpreted by assuming classical Brownian diffusion. Two parameters can then be obtained: D, the lateral diffusion coefficient and M, the mobile fraction of the diffusing molecule. When the radius of the photobleached area is small compared to the diffusion area, M must be equal to 1 for freely diffusing species. In fact, most of the data reported so far in biological membranes for transmembrane proteins shows a value of M <1. This lack in total fluorescence recovery can be interpreted as a restriction to free-diffusion behaviour. Parameters obtained then have to be re-evaluated to recognize the effect of time-dependent interactions in a field of random energy barriers.

An experimental approach to that question has been proposed by Feder et al. [15] by introducing anomalous subdiffusion in the motion of transmembrane proteins. Many sources of motion restriction can lead to anomalous diffusion (for a review, see [16,17]). Saxton has performed extensive numerical simulations to help with identifying the sources of anomalous diffusion (obstacles, binding, etc.) using SPT measurements [18,19] and he declined this more recently to FRAP experiments [20] using fractional Brownian motion (fBm) or continuous time random walk (CTRW) models as sources of anomalous diffusion.

Membrane bound proteins should also be submitted to several interactions with their surrounding environment that should account for an anomalous subdiffusion behavior. Sources of deviation from Brownian motion in their lateral diffusion may include lipid domain trapping, binding to immobile proteins and/or obstruction by cytoskeletal elements. These different possible interactions can exhibit different characteristic times or different distributions of characteristic times. Here, diffusion of an intracellular membrane-bound protein domain (pleckstrin homology (PH) domain of EFA6, the ARF6 exchange factor) has been analyzed inside living cells by FRAP experiments. Previous studies have shown that these proteins are linked to the polar head of PI(4,5)P${}_{2}$ lipids by means of electrostatic interactions [21]. Furthermore, the protein used in this study appears to have a functional requirement to be associated with the plasma membrane within cells [22,23]. In this paper, numerical simulation of the CTRW model of anomalous subdiffusion was first performed for a single spot size. Based on the quality of the fit using different analytical expression, we tested the ability to retrieve this anomalous diffusion in the simulated recovery curves first and in the experimental one afterwards. We showed that performing FRAP experiments for a single spot size did not allow for discriminating between the CTRW-induced anomalous diffusion case and the empirical classical approach using mobile and immobile fraction.

We then computed and performed experimental FRAP at variable radii. By plotting changes in the anomaly of the diffusion or in the mobile fraction as a function of the inverse of the bleached radius, as in Salomé et al. [5], we showed that it was possible to discriminate between the two models. Interestingly, we observed that the restriction to the mobility of the PH-EFA6 domain is not due to CTRW anomalous subdiffusion, but more certainly to the subcortical actin fences.

## 2. Results

### 2.1. Anomalous Subdiffusion Modeling

A way to describe the continuous time random walk subdiffusion is to start from a two-dimensional random diffusion process. A particle walks from trap to trap and spends a certain (random) time in each trap. It is characterized by the following operation:

(1) r→r+Δ;t→t+τ.

*r* and *t* are respectively the two-dimensional position and the age of the particle, where Δ is a two-dimensional random Gaussian variable with variance v=2D, and τ is the random time that the particle spends in the trap.

In our model, the particle is supposed to diffuse very rapidly between two traps. This travel time is therefore neglected (because it was not experimentally accessible). The time τ that the particle stays in a trap is supposed to have very strong fluctuations. This gives rise to the anomalous diffusion pattern.

As an example, a generic distribution is used which leads, after a while, to a standard Levy law in time:

(2) $$P_{0}\left(\tau \right)=\frac{\alpha }{{\left(1+\tau \right)}^{\alpha +1}}.$$

This distribution have been used in the same type of context by Naggle [24].

The Levy exponent α is the characteristic exponent of subdiffusive behavior. For long times, we have:

(3) $$\langle r^{2}\left(t\right)\rangle \propto t^{\alpha }.$$

When α<1, a spatio-temporal Fourier (Laplace) analysis leads to the following asymptotic (ω, *k* → 0) Green function:

(4) $$\tilde{g}\left(k,\omega \right)=\frac{1}{\omega (D_{\alpha }k^{2}\omega^{-\alpha }+1)};D_{\alpha }=D/\Gamma (1-\alpha ),$$

where ω and *k* are respectively the conjugate variables of position *r* and time *t*, where k=|k|. Notice that the solution of the inverse Laplace transform is a function of the variable $k^{2}t^{\alpha }$. It follows that the Green function is a function of the variable $x=r^{2}/t^{\alpha }$. When *x* is high enough, one can perform an approximate inverse transformation via a saddle point method:

(5) $$g\left(r,t\right)\propto \exp\left(-cst\;x^{\nu }\right)\;;\;\nu =\frac{1}{2-\alpha },\;cst:\;a\;\mathrm{known}\;\mathrm{constant}.$$

Notice that the exponent ν interpolate nicely between the Gaussian case (α=1) and the exponential case. The general solution of this type of anomalous diffusion process is then:

(6) $$\rho \left(r,t\right)=\int \rho_{0}\left(r^{\prime }-r\right)g\left(x\left(r^{\prime },t\right)\right)d^{2}r^{\prime },$$

where ρ is the probability density to find the particle at the point *r* at instant *t* and $\rho_{0}$ is the initial state.

As the Green function is a bell-shaped fast decreasing function, one approximates it by a Gaussian shape with the exact dispersion, $D_{\alpha }=D\sin\left(\pi \alpha \right)/\left(\pi \alpha \right)$, which can be calculated from Equation (4). This permits constructing an analytical expression of the fluorescence recovery using standard properties of Gaussian functions.

Starting from Axelrod [13], the initial spatial density, as it is immediately after a Gaussian profile laser beam extinction of waist *w*, is indeed:

(7) $$\rho_{0}\left(r\right)=\exp\left(-K\exp\left(-2\frac{r^{2}}{w^{2}}\right)\right).$$

(*K* = photobleaching constant, depending on experimental conditions [13])

Once integrated upon a disk of radius R, and after normalization to the surface of the disk and using the standard properties of the Gaussian shape in the convolution operation, one can obtain the time evolved result as a series of *n* terms. One then obtains the FRAP signal with the following equation:

(8) $$I_{R}\left(t\right)=1+\sum_{n=1}^{\infty }\frac{{\left(-K\right)}^{n}}{n!}\frac{1}{2n}\left(1-\exp\left(-\frac{2nR^{2}}{R^{2}+4nD_{\alpha }t^{\alpha }}\right)\right).$$

This function will be used to fit experimental data with *n* = 19.

Systematic corrections of this procedure are determined using numerical Monte Carlo simulations of the fluorescence recoveries, using known α and $D=D_{\alpha }\frac{\left(\pi \alpha \right)}{\sin(\pi \alpha )}$.

In order to keep in our calculation the finite size effects, the simulations were made in a ring of a radius of 30 arbitrary unit (a.u.) length explored by $10^{7}$ particles for each recovery. Radii varying from 0.5 to 3 a.u. were photodestructed during the simulation. The reflective type of boundary conditions were used. This means that, when a particle gets out of the 30 a.u. radius, it is reintroduced in the same direction at a small distance from the boundary. See Appendix A for examples of recovery curves generated numerically by this approach.

### 2.2. Validating Numerical Simulation and Analytical Models

**Figure 1:**
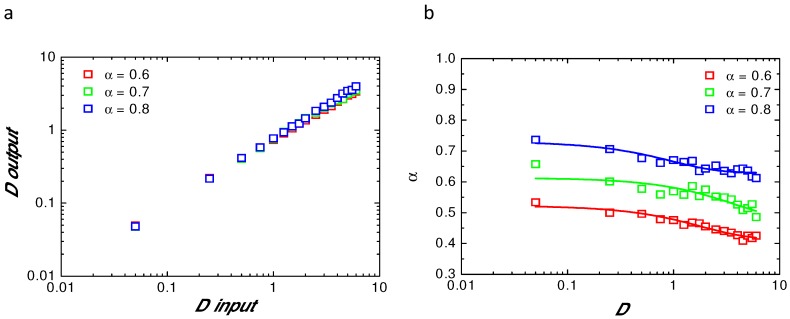
Values of the parameters(D,α) obtained from the fit of the simulated recoveries; (**a**)—*D* values obtained after fit of the fluorescence recovery with Equation (8) ($D_{output}$) as a function of *D* values used in the numerical simulation ($D_{input}$) for different α. Note that the slope is always less than 1; (**b**)—values of α obtained after fit of the simulated curves with Equation (8) for different α used in the simulation and as a function of the *D* values used in the simulation. Note that the original α value used in the simulation is never reached by fitting of the simulated recoveries.

In order to verify the validity of our analytical model, a set of numerically simulated recovery curves using anomalous diffusion as a model has been fitted with Equation (8). Each parameter (α and *D*) was tested. Figure 1a shows the value of *D* obtained after fitting of the numerical ($D_{output}$) simulation using given *D* ($D_{input}$) for the three different α tested above. Figure 1b illustrates the variation of fitted α as a function of *D* used in the simulation, for α = 0.6 (red); α = 0.7 (green); and α = 0.8 (blue). This clearly shows that both parameters (α, *D*) are always underestimated when fitting with an analytical model the numerically simulated fluorescence recoveries.

This is mainly due to the finite size and finite time effect of our numerical simulations and paradoxically is also nicely mimicking what could occur experimentally in a finite size cell reservoir.

### 2.3. Challenging Analytical Models to Identify Numerically Simulated Anomalous Diffusion Fluorescence Recoveries

Fluorescence recoveries have been numerically simulated using CTRW anomalous diffusion as the model of molecular motion. These curves were then fitted with three different analytical expression of FRAP recoveries, each being specific to a diffusion model:

- Anomalous diffusion motion (aDm): see Equation (8) in Section 2.1,
- Free Brownian motion (Bm):
  (9) $$I_{R}\left(t\right)=\sum_{1}^{\infty }\frac{{\left(-K\right)}^{n}}{n!}\frac{1}{1+n+\frac{8nDt}{R^{2}}},$$
- Restricted Brownian motion (rBm):
  (10) $$I_{R}\left(t\right)=\left(1-M\right)\frac{1-e^{-K}}{K}+M\sum_{1}^{\infty }\frac{{\left(-K\right)}^{n}}{n!}\frac{1}{1+n+\frac{8nDt}{R^{2}}},$$
  where M accounts for the mobile fraction.

**Figure 2:**
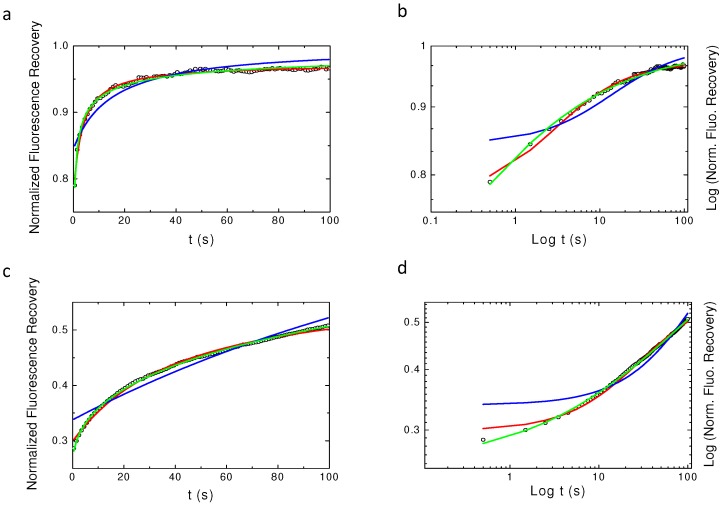
Best fits using the different models of normal and log–log plots of simulated continuous time random walk CTRW anomalous sub-diffusion recoveries. α=0.6 is the value used for the simulation in the four plots. In blue, the free Brownian motion Bm model (Equation (9)), in red restricted Brownian motion rBm model (Equation (10)) and in green anomalous diffusion motion (aDm) (Equation (8)) fits. In (**a**,**b**), *D* = 2 and, in (**c**,**d**), *D* = 0.1. (**a**,**c**) Are the normal plots while (**b**,**d**) are the log–log plots. From these graphs, it can be seen that one can hardly distinguish between the rBm model (red) and the aDm model (green) fits of the simulated recovery.

Figure 2 shows the obtained results for the three tested models (aDm in green, Bm in blue, rBm in red) with two different values of $D_{\alpha }$ and with α = 0.6. It can easily be seen that the Bm does not fit to the curve, as expected, but surprisingly it can also be seen that aDm and rBm models fit quasi equivalently the numerical simulation. Even a log–log plot (Figure 2b,d) hardly allows for directly separating the two models. Still, log–log plots show that these models can be discriminated at short times (t $\ll \tau_{c}$), $\tau_{c}$ being the characteristic half-time of the recovery) and at very long times (t $\gg \tau_{c}$).

### 2.4. Single Spot Fluorescence Recovery After Photobleaching Does Not Allow for Identifying the Nature of PH-EFA6 Diffusion in Cells

**Figure 3:**
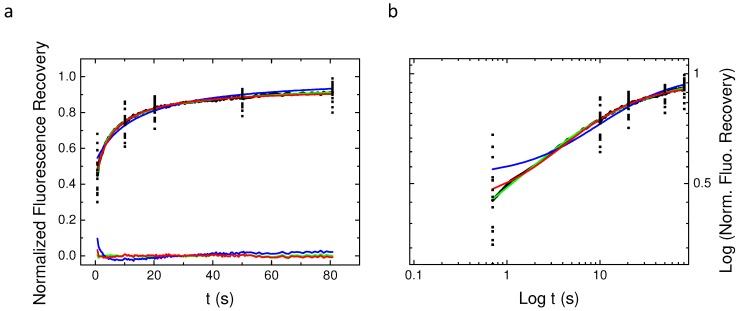
Average The three models Bm, rBm and aDm) were used to fit the average recovery curve obtained from 45 different experiments. The normal (**a**) plot shows the residual from the fit of the different models. Note that only the Bm model fit is inaccurate. The log–log plot (**b**) illustrates again the difficulty to discriminate between the aDm and the rBm model in the goodness of the fit.

Fluorescence recovery after photobleaching experiments have been performed on 15 different Baby Hamster Kidney (BHK) cells (three recoveries per cell on average) expressing the PH domain of EFA6 linked to the Green Fluorescent Protein(GFP). These data have been acquired at a given radius using the 63× objective (see the experimental section for explanation). EFA6, an exchange factor for ARF 6 (a small G protein),has been described as being located on the internal part of the plasma membrane by means of its PH domain interaction with PI(4,5)P${}_{2}$ lipids [23]. Figure 3 shows the average fluorescence recovery (black line, mean of the 45 recoveries) as well as some points extracted from of the 45 different recoveries in order to illustrate the discrepancy observed when working with cells. This mean fluorescence recovery has been fitted by the three different models used in the previous section. On the bottom of Figure 3a is depicted the differences between the fit and the observed fluorescence ($F_{f}-F_{o}$) in order to illustrate the quality of the fit. From Figure 3a (normal plot) and Figure 3b (log-log plot) it can easily be seen that except for the free Brownian motion model (Bm), the nature of the diffusion of PH-EFA6 cannot be discriminated between restricted Brownian motion (rBm) and anomalous diffusion (aDm). This is confirmed by a $\chi^{2}$ statistical test to probe the quality of the fit as shown in Table 1. Table 1 also summarized the average values of the set of parameters (*D*, M, $D_{\alpha },\;\alpha$) that can be extracted from the different fits.

### 2.5. Variable Radii Fluorescence Recovery After Photobleaching Allows Correct Estimation of the Anomalous Sub-Diffusion Exponent α

**Figure 4:**
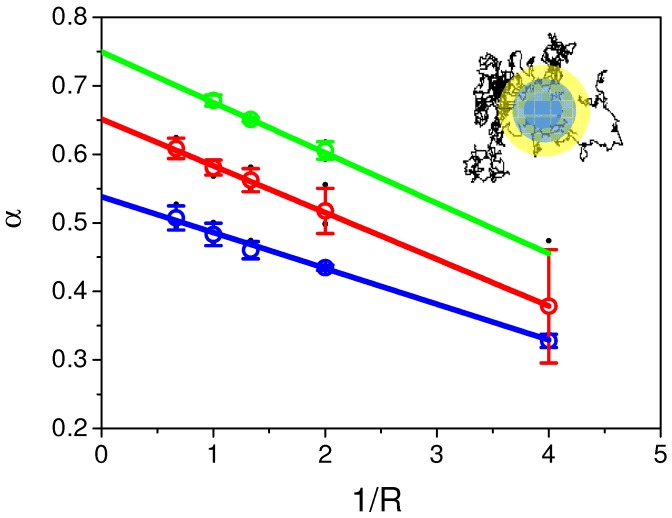
Values of α obtained from the fit of Continuous Time Random Walk model simulated recoveries as a function of 1/R. Values of α introduced in the fit were respectively 0.6 (in blue), 0.7 (in red) and 0.8 (in green). Dots represent the mean ± s.d. values of α obtained with the fit using Equation (8) of simulated recoveries for a set of *D* values (0.01, 0.05, 0.1, 0.5, 1, 2, 3, black dots in the graph). Extrapolation at 1/R = 0of the linear fit of the different α obtained from the fits of recoveries at different radii gives α values close to the one used for the simulations.

Previous results using direct analysis on both numerically simulated recoveries and experimental recoveries clearly showed that: (i) parameters α and $D_{\alpha }$ of the aDm model were always underestimated; (ii) aDm and rBm models could only be discriminated at short and long times. Nevertheless, since time and space are correlated in diffusion and since experimental time-scale is finite, variable radii FRAP experiments were firstly numerically simulated and performed after on cells (see the experimental section for explanation). Each parameter (α and $D_{\alpha }$) were estimated again, by fitting simulated recoveries at different radii (R) (see Monte Carlo simulation section for explanation) with our analytical model (Equation (8)). Figure 4 shows the behavior of fitted α as a function of 1/R Theory of anomalous diffusion processes predicts that α is space-invariant in a “homogeneously heterogeneous” environment. It can be directly seen in the plot that this is not what our data suggest, but, on the contrary, they showed that a linear dependence of α as a function of 1/R (at least for R > 1 a.u.) with a negative slope is observed for the three tested values of α (α = 0.6 in blue, α = 0.7 in green, α = 0.8 in red). Intercept of this linear regression (R→∞) leads to values closed to the input α in the numerical simulation.

**Figure A2:**
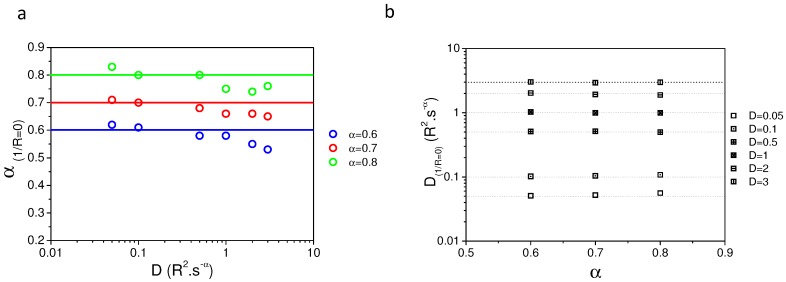
Values of α (**a**) and *D* (**b**) at 1/R=0 intercepts.

Similar results were obtained when plotting *D* fitted as a function of 1/R. Therefore, in order to test the hypothesis that α and *D* could be correctly determined by performing linear regressions of fitted values of both parameters as a function of 1/R, a set of numerical simulations were performed using different *D*, α and R. Values of α and *D* at 1/R =0 intercepts are resumed in Appendix Figure A2. Figure A2a,b show that, except for few values, when using this approach, α and *D* can be estimated with an error of less than 5% of their real values. With regard to the discrepancy of the experiments on cells, this uncertainty seems accurate enough for correct determination of both parameters in the case of anomalous diffusion processes using variable radii.

### 2.6. Continuous Time Random Walk Anomalous Subdiffusion Does Not Explain PH-EFA6 Motions in the Plasma Membrane of BHK Cells

Variable radii FRAP have already been proposed by Salomé et al. in order to characterize membrane domains in cells [5]. They found that both numerical simulation and experimental approaches that fit recovery curves using the rBm model led to a linear regression of the mobile fraction (M) as a function of 1/R with a positive slope. In this study, our results show that the same approach is valid with a aDm model and that plotting of α as a function of 1/R led to a linear regression with a negative slope. Therefore, several experiments (*n* < 30) have been performed on cells expressing the PH domain of EFA6 at variable radii of photodestruction (see Table 2 for the values of the different radii). Experimental recoveries were fitted with both models (aDm and rBm).

**Figure 5:**
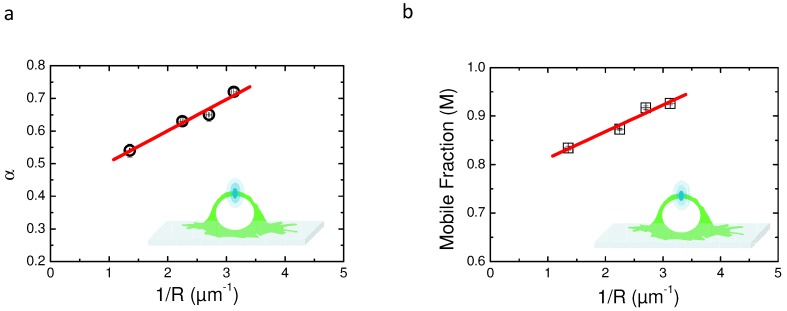
Comparison of the two models aDm and rBm using variable radii FRAP (vr-FRAP) in the case of PH-EFA6 diffusion in the plasma membrane of BHK cells. (**a**) plot of α values obtained by fitting the experimental recoveries with Equation (8) as a function of 1/R. The plot exhibit a positive slot in opposite to the one observed in Figure 4, suggesting an absence of a CTRW anomalous subdiffusion in the motion of PH-EFA6; (**b**) plot of M value obtained by fitting the experimental recoveries with Equation (10) as a function of 1/R. The plot exhibits a positive slope as observed in [5,25], suggesting a diffusion with trapping in spatial domains.

Figure 5 shows the behavior of the characteristic parameters of each model (α for aDm and M for rBm) as a function of 1/R. Figure 5a depicts the linear dependence of experimental α as a function of 1/R. These results clearly show a positive slope for the regression, suggesting that the aDm model is not the correct model for the analysis of diffusion in the case of PH-EFA6 in this experimental time and length scale. On the contrary, Figure 5b, where M is plotted as a function of 1/R, clearly shows a positive slope as already observed in Salomé’s work [5]. This shows that, in the case of PH-EFA6 diffusion in the plasma membrane of BHK cells, the rBm is the more appropriate model to describe the restriction of diffusion observed in FRAP experiments.

Schram et al. empirically determined a relationship between the size of the trapping domains (L) and the variation of the mobile fraction (M) [25]:

(11) $$M=M_{p}+\frac{0.63.L}{R};L<R.$$

Using Equation (11), we could determine that 75% of PH-EFA6 molecules exhibit free diffusion while the 25% left are confined in domains of approximately a 90 nm radius.

## 3. Discussion

This work has been initiated to characterize the nature of the diffusion of molecules binding the inner leaflet of the cell plasma membranes by means of FRAP experiments. In a first attempt, we decided to compare experimental data obtained with the PH domain of EFA6 expressed in BHK cells to FRAP curves generated from anomalous subdiffusive particles numerically simulated. Then, we analyzed the recoveries with three different diffusion models, namely the free Brownian motion (Bm), the restricted Brownian motion (rBm) and the CTRW anomalous subdiffusion (aDm). Four parameters can be extracted from these diffusion models. The Brownian diffusion coefficient *D* and the mobile fraction M (M = 1 in the case of Bm) on one side, and the anomalous subdiffusion exponent α and its related anomalous diffusion coefficient $D_{\alpha }$ on the other side. The aDm model has been extensively studied by numerical simulations. Direct analysis of numerically simulated curves lead to an underestimation of $D_{\alpha }$ and α. This was already observed by Feder et al. who proposed, in order to circumvent this underestimation, to add a mobile fraction (M) as a new parameter [15]. On a physical point of view, this is incorrect since the phenomenological parameter “mobile fraction” is indeed a part of α as discussed by Nagle et al. [24]. This underestimation of $D_{\alpha }$ and α is mainly due to a finite size effect (space and time) that cannot be easily overcome either in simulations or in experiments. We directly tested for anomalous subdiffusion in the simulated and experimental curves by fitting the recovery curves with normal and anomalous equations and look for systematic deviations of the fit, both in linear plots to see the fit at large times and log-log plots to see the fit at short times. From this approach, we could see that the Bm can be immediately discarded. The difference between the aDm and the rBm could only be observed at very short times (log-log plots) and very long times. Unfortunately, these two extreme times are hardly easy to analyze in experiments. Indeed, at short times, the curve may be distorted by diffusion during the bleach pulse [26] and limits in the frequency of data collection. At long times, motion of the membrane or photobleaching of the fluorescent probe might appear. This is illustrated here in our experimental data. Fits of single spot fluorescence recoveries did not allow for determination without uncertainties which of the aDm or the rBm model reflect the nature of PH-EFA6 diffusion in the plasma membrane of BHK cells. Although underestimated, the α value we found here, when fitting with the aDm model, reflect a strong deviation from the Brownian motion and suggest that PH-EFA6 explores a strongly compartmentalized landscape while traveling in the inner leaflet of the plasma membrane. Nevertheless, this α value, as well as the M value in the case of the rBm model, are higher than the one found for the IgE receptor transmembrane protein in RBL cells (α=0.46±0.22) [15]. Using single particle tracking experiments, other transmembrane proteins such as MHC class I in HeLa cells have also been shown to exhibit anomalous subdiffusion with an α value close to 0.5 [27]. On the contrary, other transmembrane proteins exhibit high α values (α = 0.8) (Kv2.1 potassium channel in HEK293T cells [28]) or pure Brownian motion (MHC class II in CHO cells [29] or aquaporin-1 in MDCK cells [30].

The inability of FRAP to cover several decades of time as SPT or FCS techniques will do can be overcome by probing the environment at different space scale using variable radii FRAP [5,6]. Here, we have simulated recoveries in the case of CTRW anomalous subdiffusion at different space scales and fit them with the aDm model in order to extract the set of parameters (α, *D*). By monitoring the change of fitted (α, *D*) parameters as a function of space (1/R), we observed that the fitted values of α decreased with an increasing radius of observation. This was an unexpected result, since, in our CTRW model, the anomalous sub-diffusion exponent α is supposed to be spatially invariant. Nevertheless, this could be explained by finite size effects (finite space and time used in our numerical simulations). We showed that, in the case of the CTRW model, the correct values of α and *D* could be determined at 1/R = 0, i.e., when R →∞. This is one way to overcome the finite size effect of the simulations.

Then, we applied this approach to the experimental recoveries obtained at different radii. Surprisingly, we observed the opposite tendency to the one observed in our simulation, suggesting that the CTRW anomalous subdiffusion is not the correct model to describe the motion of PH-EFA6 in BHK cells. On the contrary, when monitoring the change of the mobile fraction obtained by fitting the experimental recoveries with the rBm model, we observed the same tendency as the one described in [5,31], i.e., an increase of the mobile fraction with a decreasing radius. Using this approach, we could determine that 25% of PH-EFA6 molecules are confined in domains of a 90 nm radius.

As stated in the introduction, CTRW is not the only source of anomalous subdiffusion. The increase of the experimentally determined α with a decreasing radius can also be an apparent consequence of a crossover regime with two different diffusion coefficients as it is described by the rBm model in this study. Using FCS experiments and simulations at different radii of a two-phase, two component lipid mixtures at different temperatures, Favard et al. showed that changes in an anomalous subdiffusion exponent α could nicely predict the phase transitions temperatures but failed in determining the average size of domains coexisting in the two phases [2]. On the contrary, by monitoring the change in diffusion regimes, they could nicely determine the mean size of the gel-phase domains. If we extend this approach to our α plot as a function of the probe’s radius, we see that the transition from anomalous subdiffusion (α<1) to normal diffusion (α=1) occurs at a radius of 160 nm, i.e., not far from the values obtained with the rBm model.

The range of domain sizes observed here (90 to 160 nm radius), independently from the model used to describe the dynamics of PH-EFA6, is likely to be due to subcortical actin cytoskeleton. Equivalent sizes have been observed in NRK cells [32] using electron microscopy, and recently in several cell lines, by monitoring membrane lipids dynamics using STED-FCS [33]. Interestingly, Krapf et al. described that this meshwork has a fractal dimension and could therefore lead to anomalous subdiffusion [34]. Therefore, further investigations and numerical simulations using a meshwork with a fractal dimension as the origin of the anomalous subdiffusion are likely to be conducted in order to understand the origin of our α=f(1/R) behavior in our vrFRAP experiments.

## 4. Materials and Methods

### 4.1. Monte Carlo Simulation

In order to keep in our calculation the finite size effects, the simulations were made in a ring of a radius of 30 arbitrary unit (a.u.) length explored by 10${}^{7}$ particles for each run. Radii varying from 0.5 to 3 a.u. were photo-destructed. A reflective type of boundary conditions was used. This means that, when a particle gets out of the 30 a.u. radius, it is re-introduced in the same direction at a small distance from the boundary.

### 4.2. Cell Culture and Transfection

Baby hamster kidney cells (BHK) were grown on a coverslip in BHK-21 medium(Thermo-Fisher Scientific, Waltham, MA USA 02451), containing 5% Fetal Calf Serum, 10% tryptose phosphate broth, 100 U/mL penicillin, 100 μg/mL stretomycin and 2 mM l-glutamine. Cells were transfected using Fugene 36 h before the FRAP experiments with a pC1EGFPPHEFA6 plasmid. Fugene containing medium was replaced 12 h before the experiments by fresh medium. pC1EGFPPHEFA6 contains the sequence for both PH-EFA6 domain and Enhanced GFP (EGFP). as a fluorescent label, linked to the N-terminus of the PH-EFA6 domain in order to avoid any perturbation to the membrane linkage.

### 4.3. Fluorescence Recovery After Photobleaching Experiments

Fluorescence Recovery After Photobleaching measurements were made with a commercially available confocal microscope, Leica TCS-SP1 (Leica Microsystems, Heidelberg, Germany). Prebleached images were firstly acquired to ensure for the lack of photo-destruction during the observation. A brief laser pulse (200 ms) was then delivered to the cell on a given and fixed position. Images were thereafter recorded at given intervals (440 ms) using a spectral window for fluorescence emission between 500 and 600 nm. The intensity ratio between the extinction laser beam and the monitoring laser beam was fixed to $10^{6}$. Each fluorescence recovery was recorded for 100 s at 25 ${}^{\circ }$C, containing 150 experimental values (the recovery curve was sampled every 0.44 s in the beginning and 1 s in the end to avoid photobleaching during the monitoring). Focusing the laser by the microscope objectives produced a Gaussian intensity distribution of the beam in the object plane. This distribution was monitored using a di-palmitoyl phosphatidyl-choline (DPPC lipid multilamellar preparation at 25 ${}^{\circ }$C (T < Tm) labelled with 1-palmitoyl-2-6-[(7-nitro-2- 1,3-benzoxadiazol-4-yl)amino]hexanoyl-sn-glycero-3-phosphocholine (NBD-PC) (1%mol:mol). Since no diffusion occurs at this temperature, the image obtained immediately after the end of the bleaching pulse shows a hole in the fluorescent preparation that allows for measurement of the radius of the disk bleached (R) and determination of the shape of the intensity profile in the *x*,*y* plane. These measurements were confirmed by the use of fluorescent beads with a maximum wavelength of emission at 500 nm [35]. The following values were obtained for the waist as a function of the objective used:

## 5. Conclusions

In conclusion, by performing FRAP at variable radii, we found a way to discriminate that CTRW induces anomalous subdiffusion from other restricted motion in the plasma membrane of living cells. We also show that, while traveling in the inner leaflet of the plasma membrane, PH-EFA6 is not stopped in various traps with different residence times, but, on the contrary, is mainly freely diffusing with on average of 25% of the molecules confined in 90–160 nm radius open domains, most probably due to the actin cortical cytoskeleton.

## Figures and Tables

**Table 1:** Parameter values obtained by the fit of the average experimental recovery with the different analytical models.

Model	*D* ($\mu m\cdot s^{-1}$) ${}^{1}$	M	α	$D_{\alpha }$ ($\mu m\cdot s^{-\alpha }$) ${}^{2}$	$\chi^{2}$ ${}^{3}$
Bm ${}^{4}$	0.12 ± 0.06	1	-	-	5.7 ± 0.5
rBm ${}^{5}$	0.22 ± 0.01	0.92 ± 0.01	-	-	3.8 ± 1.6
aDm ${}^{6}$	-	-	0.65 ± 0.02	1.48 ± 0.05	2.9 ± 1.6

${}^{1}$*D*: Brownian diffusion coefficient; ${}^{2}$$D_{\alpha }$: Anomalous diffusion coefficient; ${}^{3}$$\chi^{2}$ test is used to verify the accuracy of the experimental values fit using the different models; ${}^{4}$ Bm: Free Brownian motion; ${}^{5}$ rBm: restricted Brownian motion; ${}^{6}$ aDm: anomalous diffusion model.

**Table 2:** Size of the different radii (R) measured with the objectives used in this work.

Objective	R (μm)	ΔR (μm)	Laser Waist (nm)
16× (NA ${}^{1}$ = 1.0)	0.74	0.04	370
40× (NA = 1.3)	0.44	0.03	222
63× (NA = 1.4)	0.37	0.01	185
100× (NA = 1.4)	0.32	0.01	160

${}^{1}$ NA: Numerical aperture of the objective.

**Figure A1:**
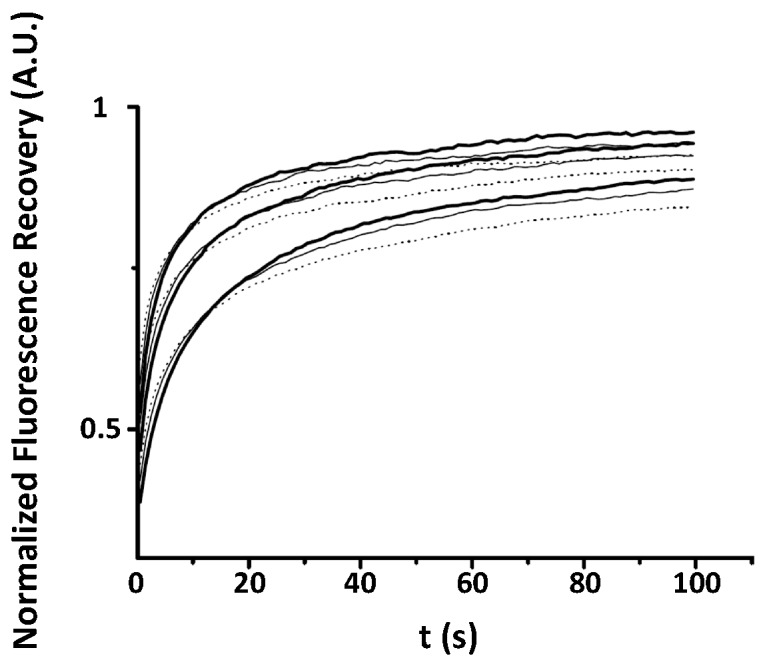
Monte Carlo simulation of normalized fluorescence recoveries in the case of CTRW anomalous subdiffusion. Different values of *D* and α have been tested in the simulations. Here, values of D=0.5;1;1.5 are represented from bottom to top with different α in each case: 0.6 (dots); 0.7 (thin line); 0.8 (thick line). The Monte Carlo simulation has been constructed with $10^{7}$ individual trajectories.

## Appendix A. Examples of Numerically Generated Fluorescence Recovery Curves

Figure A1 depicts some fluorescence recovery curves obtained with our numerical simulations for different *D* and α inserted in the simulation.

## Appendix B. Comparison of Input and Fitted D and α at Variable Radii

Figure A2 plots values of α (Figure A2a) and *D* (Figure A2b) at 1/R = 0 intercepts. Figure A2a,b show that extrapolated $\alpha_{\left(1/R=0\right)}$ and extrapolated $D_{\left(1/R=0\right)}$ are found to be with an error of less than 5% of their simulation inserted values.

## References

[1] Singer S.J., Nicolson G.L. (1972). The Fluid Mosaic Model of the Structure of Cell Membranes. Science.

[2] Favard C., Wenger J., Lenne P.F., Rigneault H. (2011). FCS diffusion laws in two-phase lipid membranes: Determination of domain mean size by experiments and Monte Carlo simulations. Biophys. J..

[3] Hac A.E., Seeger H.M., Fidorra M., Heimburg T. (2005). Diffusion in two-component lipid membranes—A fluorescence correlation spectroscopy and monte carlo simulation study. Biophys. J..

[4] Edidin M., Kuo S.C., Sheetz M.P. (1991). Lateral movements of membrane glycoproteins restricted by dynamic cytoplasmic barriers. Science.

[5] Salomé L., Cazeils J.L., Lopez A., Tocanne J.F. (1998). Characterization of membrane domains by frap experiments at variable observation areas. Eur. Biophys. J..

[6] Yechiel E., Edidin M. (1987). Micrometer-scale domains in fibroblast plasma membranes. J. Cell Biol..

[7] Lingwood D., Simons K. (2010). Lipid rafts as a membrane-organizing principle. Science.

[8] Kusumi A., Sako Y., Yamamoto M. (1993). Confined lateral diffusion of membrane receptors as studied by single particle tracking (nanovid microscopy). Effects of calcium-induced differentiation in cultured epithelial cells. Biophys. J..

[9] Sako Y., Kusumi A. (1994). Compartmentalized structure of the plasma membrane for receptor movements as revealed by a nanometer-level motion analysis. J. Cell Biol..

[10] Wawrezinieck L., Rigneault H., Marguet D., Lenne P.F. (2005). Fluorescence correlation spectroscopy diffusion laws to probe the submicron cell membrane organization. Biophys. J..

[11] Lenne P.F., Wawrezinieck L., Conchonaud F., Wurtz O., Boned A., Guo X.J., Rigneault H., He H.T., Marguet D. (2006). Dynamic molecular confinement in the plasma membrane by microdomains and the cytoskeleton meshwork. EMBO J..

[12] Eggeling C., Ringemann C., Medda R., Schwarzmann G., Sandhoff K., Polyakova S., Belov V.N., Hein B., von Middendorff C., Schönle A. (2009). Direct observation of the nanoscale dynamics of membrane lipids in a living cell. Nature.

[13] Axelrod D., Koppel D.E., Schlessinger J., Elson E., Webb W.W. (1976). Mobility measurement by analysis of fluorescence photobleaching recovery kinetics. Biophys. J..

[14] Soumpasis D.M. (1983). Theoretical analysis of fluorescence photobleaching recovery experiments. Biophys. J..

[15] Feder T.J., Brust-Mascher I., Slattery J.P., Baird B., Webb W.W. (1996). Constrained diffusion or immobile fraction on cell surfaces: A new interpretation. Biophys. J..

[16] Bouchaud J.P., Georges A. (1990). Anomalous diffusion in disordered media: Statistical mechanisms, models and physical applications. Phys. Rep..

[17] Hoefling F., Franosch T. (2013). Anomalous transport in the crowded world of biological cells. Rep. Prog. Phys..

[18] Saxton M.J. (1994). Anomalous diffusion due to obstacles: A Monte Carlo study. Biophys. J..

[19] Saxton M.J. (1996). Anomalous diffusion due to binding: A Monte Carlo study. Biophys. J..

[20] Saxton M.J. (2001). Anomalous Subdiffusion in Fluorescence Photobleaching Recovery: A Monte Carlo Study. Biophys. J..

[21] Hyvönen M., Macias M.J., Nilges M., Oschkinat H., Saraste M., Wilmanns M. (1995). Structure of the binding site for inositol phosphates in a PH domain. EMBO J..

[22] Franco M., Peters P.J., Boretto J., van Donselaar E., Neri A., D’Souza-Schorey C., Chavrier P. (1999). EFA6, a sec7 domain-containing exchange factor for ARF6, coordinates membrane recycling and actin cytoskeleton organization. EMBO J..

[23] Macia E., Partisani M., Favard C., Mortier E., Zimmermann P., Carlier M.F., Gounon P., Luton F., Franco M. (2008). The pleckstrin homology domain of the Arf6-specific exchange factor EFA6 localizes to the plasma membrane by interacting with phosphatidylinositol 4,5-bisphosphate and F-actin. J. Biol. Chem..

[24] Nagle J.F. (1992). Long tail kinetics in biophysics?. Biophys. J..

[25] Schram V., Tocanne J.F., Lopez A. (1994). Influence of obstacles on lipid lateral diffusion: computer simulation of FRAP experiments and application to proteoliposomes and biomembranes. Eur. Biophys. J..

[26] Waharte F., Brown C.M., Coscoy S., Coudrier E., Amblard F. (2005). A two-photon FRAP analysis of the cytoskeleton dynamics in the microvilli of intestinal cells. Biophys. J..

[27] Smith P.R., Morrison I.E., Wilson K.M., Fernández N., Cherry R.J. (1999). Anomalous diffusion of major histocompatibility complex class I molecules on HeLa cells determined by single particle tracking. Biophys. J..

[28] Weigel A.V., Simon B., Tamkun M.M., Krapf D. (2011). Ergodic and nonergodic processes coexist in the plasma membrane as observed by single-molecule tracking. Proc. Natl. Acad. Sci. USA.

[29] Vrljic M., Nishimura S.Y., Brasselet S., Moerner W.E., McConnell H.M. (2002). Translational diffusion of individual class II MHC membrane proteins in cells. Biophys. J..

[30] Crane J.M., Verkman A.S. (2008). Long-range nonanomalous diffusion of quantum dot-labeled aquaporin-1 water channels in the cell plasma membrane. Biophys. J..

[31] Baker A.M., Saulière A., Gaibelet G., Lagane B., Mazères S., Fourage M., Bachelerie F., Salomé L., Lopez A., Dumas F. (2007). CD4 interacts constitutively with multiple CCR5 at the plasma membrane of living cells. A fluorescence recovery after photobleaching at variable radii approach. J. Biol. Chem..

[32] Morone N., Fujiwara T., Murase K., Kasai R.S., Ike H., Yuasa S., Usukura J., Kusumi A. (2006). Three-dimensional reconstruction of the membrane skeleton at the plasma membrane interface by electron tomography. J. Cell Biol..

[33] Andrade D.M., Clausen M.P., Keller J., Mueller V., Wu C., Bear J.E., Hell S.W., Lagerholm B.C., Eggeling C. (2015). Cortical actin networks induce spatio-temporal confinement of phospholipids in the plasma membrane—A minimally invasive investigation by STED-FCS. Sci. Rep..

[34] Sadegh S., Higgins J.L., Mannion P.C., Tamkun M.M., Krapf D. (2017). Plasma Membrane is Compartmentalized by a Self-Similar Cortical Actin Meshwork. Phys. Rev. X.

[35] Schneider M.B., Webb W.W. (1981). Measurement of submicron laser beam radii. Appl. Opt..

